# Visual Sensor Networks for Indoor Real-Time Surveillance and Tracking of Multiple Targets

**DOI:** 10.3390/s22072661

**Published:** 2022-03-30

**Authors:** Jacopo Giordano, Margherita Lazzaretto, Giulia Michieletto, Angelo Cenedese

**Affiliations:** 1 Department of Information Engineering, University of Padova, 35131 Padova, Italy; margherita.lazzaretto@studenti.unipd.it (M.L.); angelo.cenedese@unipd.it (A.C.); 2 Department of Management and Engineering, University of Padova, 36100 Vicenza, Italy

**Keywords:** smart cameras, heterogeneous camera network, distributed approach, Kalman filter, tracking, pose optimization, game theory

## Abstract

The recent trend toward the development of IoT architectures has entailed the transformation of the standard camera networks into smart multi-device systems capable of acquiring, elaborating, and exchanging data and, often, dynamically adapting to the environment. Along this line, this work proposes a novel distributed solution that guarantees the real-time monitoring of 3D indoor structured areas and also the tracking of multiple targets, by employing a heterogeneous visual sensor network composed of both fixed and Pan-Tilt-Zoom (PTZ) cameras. The fulfillment of the twofold mentioned goal was ensured through the implementation of a distributed game-theory-based algorithm, aiming at optimizing the controllable parameters of the PTZ devices. The proposed solution is able to deal with the possible conflicting requirements of high tracking precision and maximum coverage of the surveilled area. Extensive numerical simulations in realistic scenarios validated the effectiveness of the outlined strategy.

## 1. Introduction

A Visual Sensor Network (VSN) is a multi-agent system constituted of a collection of spatially distributed smart cameras. In recent years, due to the ever-improving sensing and computational capabilities and the reduced cost of visual sensors, such architectures have gained popularity and are currently employed in many tasks, ranging from the more traditional surveillance and security scenarios to the cutting-edge IoT applications, e.g., in environmental monitoring, sports, and education contexts [1,2,3].

The ongoing IoT-driven incentive towards the development of cooperative distributed solutions leads to the progressive substitution of the traditional centralized VSNs made up of static devices, namely cameras having a fixed pose (i.e., position and orientation), in favor of more flexible, decentralized, and intelligent systems [4,5,6]. The modern VSNs do not generally envisage a central computing unit, but accomplish the assigned task by relying on a distributed approach. Moreover, they often include PTZ cameras, namely visual sensors having controllable pan and tilt angles and zoom parameters and, thus, characterized by variable orientations and fields of view. The introduction of these new visual sensors enhances the VSNs’ scalability and robustness and, at the same time, allows reducing both the number of cameras needed to cover a given area and the communication and computational burden imposed by data exchange. On the other hand, to take full advantage of the PTZ cameras, it is necessary to dynamically optimize their parameters depending on some external factors, including their (fixed) position, the potential occlusions, the occurrence of failures, and/or targets to follow.

In this work, the attention is focused on a VSN made up of both fixed and PTZ cameras, required to monitor a given area with the purpose of detecting and tracking one or more targets. To this aim, a decentralized solution is proposed: it entails the optimization of the PTZ parameters through the exploitation of both the network/environment topology and the tracking information shared among the same cameras.

### 1.1. Related Works

The monitoring performance of the traditional VSNs, composed exclusively of fixed cameras, uniquely depends on the device number and positioning in the considered environment (see, e.g., [7,8,9]). On the contrary, when accounting for VSNs involving also PTZ cameras, several parameters can be dynamically selected and actively controlled in order to optimize the surveillance task’s fulfillment.

As in the existing literature, the selection of the PTZ parameters can be performed in different fashions, as for instance exploiting the notions of control theory, decision theory, game theory, or resting upon learning algorithms or suitable heuristics [5,10,11]. More in detail, the parameter selection strategies based on control theory exploit a feedback mechanism to minimize the difference between the observed and desired system state. In this direction, in [12], a PID controller was designed to perform the real-time tracking of a target by means of a single PTZ camera. In [13], each movement of the pan and tilt servo camera was controlled by some signals calculated by adopting the model predictive control approach. Nonetheless, although the employment of control-based methods appears to be efficient and sound, their use may lack flexibility. The PTZ parameter selection methods based on decision theory entail the possibility of choosing among different actions even if their consequences are not perfectly known. Specifically, the Markov Decision Process (MDP) and Partially Observable MDP (POMDP) models are employed in many VSN control solutions. For example, in [14], both the MDP and POMDP approaches were adopted in the determination of the optimal configuration of several active cameras, required to maximize the number of observed targets, while guaranteeing a minimum view resolution. A particularly interesting branch of decision theory is game theory, wherein the decisions are assumed to be independent in the selection process. For this reason, this approach results in being a valuable technique when coping with real-time constraints. Indeed, it permits solving cooperative and competitive problems and dealing with non-convex and discontinuous utility functions [15]. Along this line, a game theoretic approach was presented in [16] to address the distributed pose optimization of a group of PTZ cameras. The proposed solution relies on the maximization of various performance metrics (including tracking accuracy and image resolution), with the purpose of finding the best multi-camera system configuration. Such a strategy was further developed in [4], wherein the presence of groups of targets, capable of merging and dividing, was assumed. More recently, PTZ parameter selection procedures relying on Reinforcement Learning (RL) techniques have gained popularity. For instance, a Q-learning-based solution was presented in [17] for smoothly controlling a PTZ camera. A soft actor–critic RL system was, instead, designed in [18] to perform the decentralized reconfiguration of a camera network involving PTZ sensors and devices mounted on flying platforms. The main issue related to the RL solutions consists of their intrinsic black box nature: in some contexts, it is not possible or safe to rely upon such controllers, whose performance may not be completely predictable. Finally, the adoption of some heuristics turns out to be a valid option, especially in case of real-time control requirements. In [19], for instance, a scheduling heuristic was taken into account for dealing with the cooperative monitoring of an object through a PTZ camera network. Nonetheless, the general drawback of heuristics is that they strongly rely on the experience of the users and often on lengthy and tiring tuning procedures.

Despite the specific method adopted to select the PTZ parameters, some general observations are due when accounting for VSNs composed also of dynamic cameras and required to perform both the real-time area surveillance and the targets’ tracking. First, ensuring the cameras’ field of view (FOV) overlap turns out to be advantageous in the surveillance activities, especially when also target detection and tracking are required. This is motivated by the fact that, without the full coverage of the environment, the positions of the targets need to be estimated in correspondence with the blind spots [1]. Then, the presence of multiple targets entails a tradeoff between the surveilled area coverage and the target view image resolution.

To conclude this non-exhaustive survey of the related works, many approaches to address target tracking are described in the literature. Most of them envisage the exploitation of filtering and prediction tools, as, for example particle filters, Bayesian estimation techniques, and (extended) Kalman Filters (KFs) [1]. In detail, the approaches based on the KF are extensively used when dealing with real-time applications and often require distributed computations [20,21]. For instance, in [22], a distributed KF was studied for sensor networks with a limited sensing range, and an extended version of the same approach was investigated in [16] to perform 2D tracking employing a PTZ camera network. In [23], instead, a solution based on particle filters was considered for decentralized tracking of groups of people or individuals. Finally, in [24], a decentralized framework was presented for cooperative self-localization and multi-target tracking via Gaussian filters.

### 1.2. Contributions

Accounting for a heterogeneous VSN made up of both fixed and PTZ cameras, this work presents a strategy aiming at ensuring the real-time surveillance of a structured indoor environment, as well as multi-target tracking. The outlined procedure envisages the (optimal) selection of the adjustable parameters of the dynamic devices composing the network, resting on the game theory approach proposed in [16].

With respect to [16], the novel aspects of this work derive from the focus on real-world scenarios, wherein it is desirable to limit both the costs (in terms of employed devices) and the task execution time. In [16], the study case consisted of a unique unstructured 2D environment monitored by a broad set of PTZ cameras capable of performing both the distributed multi-target tracking and the iterative optimization of their parameters. In particular, the proposed target tracking method is based on an Extended Kalman Filter (EKF), while the parameter selection rests on the iterative solution of a computationally demanding optimization problem. In this work, instead, great attention is devoted to the real-world environment. The twofold mentioned goal is, indeed, faced by accounting for a 3D scenario consisting of a structured area composed of multiple connected rooms, and the available a priori information on the physical space partition is exploited in the solution process. In addition, the considered VSN involves a limited number of (highly expensive) PTZ cameras, while including also (low-cost) static devices. Specifically, these permit still efficiently managing the transitions between different areas, thus streamlining the PTZ parameter selection. In this direction, their presence favors also network scalability since the dynamic devices’ reconfiguration can be accomplished in a parallel manner in the different rooms.

More in detail, in this work, the double monitoring and tracking problem is addressed by modeling the targets as 3D point particles whose position is characterized by a non-null uncertainty and overcoming the concept of a planar occupancy grid. The selection of the PTZ parameters implies the identification of the optimal values for both the pan and tilt angle, jointly with the zoom (when accounting for the 2D context only, the pan angle is generally considered in the PTZ parameter selection) Inspired by the game theory approach proposed in [16], we propose an update procedure for the orientation of the PTZ cameras based on the optimization of a certain utility function. In particular, this latter is defined accounting for some criteria that are new and original with respect to [16] since the intent is to reduce the computational complexity of the parameter selection process because of the real-time constraints on the task’s execution. The utility function is maximized in a distributed manner via an iterative negotiation mechanism among some PTZ devices. In particular, different from [16], the set of PTZ cameras involved in the mentioned negotiation procedure is determined based on the a priori knowledge of the environment in terms of structure and devices’ placement. Such information is further exploited to allow for the parallelization of the parameter selection by multiple independent groups of PTZ cameras.

The principal advantage of the solution proposed in this work consists of its versatility and flexibility. Indeed, by conveniently choosing the weights that regulate the contributions in the utility function, it is possible to prioritize the monitoring task with respect to the tracking task, or vice versa. Along this line, the results of the conducted simulative campaign demonstrated its effectiveness in handling the tradeoff between the tracking precision and the image resolution, especially in the critical scenarios. Moreover, the heterogeneous nature of the network and the outlined distributed approach allow the parallelization of the parameter selection and tracking tasks, resulting in a framework that can be easily scaled up to larger and more complex environments.

As a final remark, we emphasize that, although the problems related to targets’ detection and partial/complete loss are not directly taken into account in this work, some possible actions to face these issues are discussed.

### 1.3. Paper Structure

This paper is organized as follows. In Section 2, after the problem statement, the application scenario is described and modeled. In Section 3, the elements necessary to perform real-time multi-target tracking are outlined. In particular, Section 3.1 illustrates the distributed tracking algorithm based on the EKF solution, while Section 3.2 discusses the PTZ parameter selection. After that, Section 4 presents the application environment employed to validate the strategy outlined in the previous sections, and Section 5 reports the results of the multiple test scenarios considered. In Section 6, a discussion is provided about interesting aspects revealed from the simulation results, together with possible future improvements. Finally, Section 7 reports a summary of the study and contains some final considerations.

## 2. Problem Statement, Models, and Assumptions

This section aims at illustrating the application scenario taken into account in this work: a structured and cluttered indoor environment monitored by a heterogeneous VSN. We highlight the considered twofold goal, stating the problem and discussing the models and the assumptions adopted in the design of the proposed solution.

### 2.1. Problem Statement

In this work, the attention is focused on a structured indoor 3D environment $E\in R^{3}$ composed of $n_{R}\geq 1$ *rooms* and characterized by $n_{A}\geq 1$ access points. This is supposed to be monitored by a VSN made up of $n_{C}\geq 2$ cameras, divided into $n_{S}\geq 1$ *static* visual sensors, i.e., fixed cameras, and $n_{D}\geq 1$ *dynamic* visual sensors, namely PTZ cameras. In turn, the fixed cameras are split into $n_{SHR}\geq 0$ *high-resolution* visual sensors and $n_{SWA}\geq 0$ *wide-angle* visual sensors.

In this context, we aimed at proposing an effective strategy to fulfill a twofold goal: to ensure the real-time surveillance of the described environment and to guarantee the efficient tracking of $n_{T}\geq 1$ targets, free to move in the supervised area.

### 2.2. Environment Modeling

To cope with the aforementioned goal, we define the following sets:

- The *environment access points* set $A=\{A_{1}\ldots A_{n_{A}}\}$, consisting of a single element in the currently considered setup where $n_{A}=1$;
- The *physical environment partitions* set $R=\{R_{1}\ldots R_{n_{R}}\}$ with $R_{h}\subset R^{3}$ denoting the *h*-th room composing the considered environment,
- The *high-resolution* and *wide-angle static visual sensors* set $C^{SHR}=\left\{C_{1}^{SHR}\ldots C_{n_{SHR}}^{SHR}\right\}$ and $C^{SWA}=\left\{C_{1}^{SWA}\ldots C_{n_{SWA}}^{SWA}\right\}$, respectively;
- The *dynamic* and *static visual sensors* set $C^{D}=\left\{C_{1}^{D}\cdots C_{n_{D}}^{D}\right\}$ and $C^{S}=\left\{C_{1}^{S}\cdots C_{n_{S}}^{S}\right\}$, respectively, of which this last one is the direct sum of $C^{SHR}$ and $C^{SWA}$;
- The *visual sensors* set $C=\{C_{1}\ldots C_{n_{C}}\}$ resulting from the direct sum of $C^{D}$ and $C^{S}$;
- The *targets* set $T=\{T_{1}\cdots T_{n_{T}}\}$.

In addition, we also introduce the *virtual environment partitions* set $P=\{P_{1}\ldots P_{n_{P}}\}$ and the *handout zones* set $H=\{H_{1}\ldots H_{n_{H}}\}$. The former set consists of $n_{P}\geq n_{R}$ virtual partitions of the supervised environment; formally, we have that $P_{k}\subset R^{3}$, ${⋃}_{k=1}^{n_{P}}P_{k}={⋃}_{h=1}^{n_{R}}R_{h}=E$, and $P_{k}\cap P_{\kappa }=\emptyset$ (disjoint sets) with $k,\kappa \in \{1\ldots n_{P}\},k\neq \kappa$. We emphasize that each element of P can either correspond to a physical room ($n_{P}=n_{R}$) or a part of a physical room ($n_{P}>n_{R}$). On the other side, the handout zones set is composed of $n_{H}\geq n_{P}$ environment portions corresponding to the transition areas between two adjacent virtual partitions. From a mathematical perspective, it thus holds that $H_{p}\subset R^{3}$, $H_{p}\cap H_{\rho }=\emptyset$ with $p,\rho \in \{1\ldots n_{H}\},p\neq \rho$ and $H_{p}\subseteq \left(P_{k}\cup P_{\kappa }\right)$ being $P_{k},P_{\kappa }$ adjacent virtual partitions. In particular, hereafter, we use the notation $H_{k\kappa }\left(=H_{p}\right)$ to indicate the handout zone between the *k*-th and the κ-th virtual partition; more specifically, we have that $H_{k\kappa }=H_{k\kappa }^{k}⊕H_{k\kappa }^{\kappa }$, being $H_{k\kappa }^{k}=H_{k\kappa }\cap P_{k}$ and $H_{k\kappa }^{\kappa }=H_{k\kappa }^{\kappa }\cap P_{\kappa }$ with $k,\kappa \in \{1\ldots n_{P}\},k\neq \kappa$.

All the introduced sets are listed in Table 1, where we also report the assumptions about their cardinality, many of which derive from the following statements regarding the considered scenario.

- The high-resolution fixed cameras were used *only* to monitor the access points in order to guarantee the quick and effective target detection when they enter in the environment, thanks to their increased performance, but also because of their cost. Thus, it holds that $n_{SHR}=n_{A}$;
- Characterized by ample FOVs at the cost of low resolution, the wide-angle fixed cameras were instead exploited to ensure the *best coverage*. Hence, we assumed that at least a visual sensor in the set $C^{SWA}$ is placed in each virtual partition, and in particular, this is located in order to monitor the related handout zones. Consequently, it follows that $n_{SWA}\geq n_{P}$;
- Finally, the PTZ cameras were employed to enhance the VSN *target tracking* capabilities. Therefore, it is reasonable to assume that $n_{D}\geq n_{P}$.

At the same time, we highlight that any assumption is stated as regards the specific camera’s placement within the environment: several existing algorithms allow determining the best sensor location [9,25,26,27].

### 2.3. Targets’ Modeling

Adopting a control system approach, we modeled any target as a *point particle* acting in 3D space, thus characterized by a (time-varying) position in the global inertial frame $F_{W}$, hereafter termed the *world frame*. Formally, the position of any *j*-th target, $j\in \{1\ldots n_{T}\}$, at time *t* is identified by the vector $p_{j}\left(t\right)\in R^{3}$.

Assuming then a first-order dynamics for all the targets, we have that the *j*-th target state at time *t* is described by the vector $x_{j}\left(t\right)={\left[\begin{array}{cc} p_{j}\left(t\right) & {\dot{p}}_{j}\left(t\right) \end{array}\right]}^{⊤}\in R^{6}$, stacking its position and velocity in $F_{W}$. Moreover, inspired by [16], we assumed that the introduced state evolves according to the following discrete-time dynamics with sampling time $T\in R^{+}$:

(1) $$x_{j}\left(t+1\right)=A_{j}x_{j}\left(t\right)+w_{j}\left(t\right),\;A_{j}=\left[\begin{array}{cc} I_{3\times 3} & TI_{3\times 3}\\ 0_{3\times 3} & I_{3\times 3} \end{array}\right]\in R^{6\times 6}$$

where $0_{3\times 3}\in R^{3\times 3}$ and $I_{3\times 3}\in R^{3\times 3}$ denote the square null and identity matrix, respectively. The vector $w_{j}\left(t\right)\in R^{6}$ in (1) represents an additive Gaussian noise; in particular, we assumed that $w_{j}\left(t\right)∼N\left(0_{6},W_{j}\right)$ with $0_{6}\in R^{6}$ identifying the zero mean vector and $W_{j}\in R^{6\times 6}$ representing the *j*-th target (known) covariance matrix. The point particle modeling assumption, even if it appears simplistic, allows capturing the basic behavior of a moving subject and focusing on other aspects of interest such as the coordination and cooperation among the VSN devices. We further emphasize that, although depending on the specific case, it is generally possible to relate the output of any object detection algorithm to the assumed representation. For example, if a detected object is modeled by a bounding box, then the center of such a box can be exploited in the point particle model. The uncertainty characterizing this operation can be included in a comprehensive error term affecting the cameras’ observations.

### 2.4. Cameras Modeling

In this work, every camera composing the given VSN was modeled as a *rigid body* having (possibly time-varying) position and orientation, i.e., a pose, in the world frame. Note that these quantities are often referred to as *camera extrinsic parameters* in the literature. In detail, we denote by $F_{B}$ the local frame in-built with the device so that the *x*-axis points upward, the *y*-axis points to the right, the *z*-axis points forward, and it is aligned with the device optical axis. Then, the *i*-th camera (time-invariant) position in $F_{W}$ is identified by the vector $t_{W,i}\in R^{3}$, while its orientation with respect to the world frame is represented by the rotation matrix $R_{W2B,i}\left(t\right)\in SO\left(3\right)$, (potentially) depending on the time *t*. In particular, we assumed that $R_{W2B,i}\left(t\right)$ results from the composition of three subsequent rotations around the axes of $F_{B}$, namely $R_{W2B,i}\left(t\right)=R_{z}\left(\gamma_{i}\left(t\right)\right)R_{y}\left(\beta_{i}\left(t\right)\right)R_{x}\left(\alpha_{i}\left(t\right)\right)$ with $\alpha_{i}\left(t\right),\beta_{i}\left(t\right),\gamma_{i}\left(t\right)\in \left[-\pi ,\pi \right]$.

For all the static visual sensors, the orientation is fixed and constant over time, namely $R_{W2B,i}\left(t\right)=R_{W2B,i}$, $\forall i\in \{1\ldots n_{S}\}$. On the other hand, the dynamic visual sensors are characterized by a partially time-varying orientation. Indeed, a PTZ camera can modify its orientation through a pan and/or a tilt movement, namely through a rotation around the *x*-axis and/or the *y*-axis of its $F_{B}$ of a certain controllable pan and/or tilt angle, respectively. From a mathematical perspective, we have that $R_{W,i}\left(t\right)=R_{W,i}\left(\alpha_{i}\left(t\right),\beta_{i}\left(t\right)\right)$, $\forall i\in \{1\ldots n_{D}\}$, with $\alpha_{i}\left(t\right)$ and $\beta_{i}\left(t\right)$ hereafter referred to as the *i*-th camera pan and tilt angle, respectively.

Without loss of generality, some standard assumptions were made also on the *intrinsic parameters* of all the considered visual sensors: for any camera, the focal length *f* was assumed to be unitary; no distortion was taken into account; the FOV is defined by a pair of angles affecting its height and width. We remark that the PTZ cameras can also dynamically vary their zoom settings; hence, these are characterized by three controllable degrees of freedom. Hereafter, the zoom parameter of the *i*-th dynamic visual sensor, $i\in \{1\ldots n_{D}\}$, is referred to as $\zeta_{i}\geq 0$. In addition, we took into account the maximum distance at which a target can be detected with a satisfying quality level. Hereafter, this is associated with a *minimum pixel density* value. More in detail, for any camera, we assumed computing the pixel density characterizing the FOV at the distance (along the optical axis) of a certain target: when such a density is lower than a minimum threshold, the considered target is considered as not visible.

Then, when the *j*-th target, $j\in \{1\ldots n_{T}\}$, is observed by the *i*-th visual sensor, $i\in \{1\ldots n_{C}\}$, at time *t*, its position is projected on the camera image plane. Formally, introducing the (nonlinear) function $h\left(\cdot \right):R^{3}\mapsto R^{2}$ mapping any vector $x={\left[\begin{array}{c} x_{1}\;x_{2}\;x_{3} \end{array}\right]}^{⊤}\in R^{3}$ into its projection onto the 2D plane $h\left(x\right)={\left[\begin{array}{c} x_{1}/x_{3}\;\;x_{2}/x_{3} \end{array}\right]}^{⊤}\in R^{2}$, we have that the position $z_{ij}\left(t\right)\in R^{2}$ of the *j*-th target into the *i*-th camera image plane evolves as follows:

(2) $$z_{ij}\left(t\right)=h\left(R_{W2B,i}\left(t\right)\left(\left[\begin{array}{cc} I_{3\times 3} & 0_{3\times 3} \end{array}\right]x_{j}\left(t\right)-t_{W,i})\right)\right)+v_{ij}\left(t\right)$$

where the vector $v_{ij}\left(t\right)\in R^{2}$ represents the addictive noise deriving from the projection and measurement errors for the *i*-th camera. We assumed that $v_{ij}\left(t\right)∼N\left(0_{2},V_{i}\left(t\right)\right)$ with $0_{2}\in R^{2}$ and $V_{i}\left(t\right)\in R^{2\times 2}$; in particular, the covariance matrix was modeled as a diagonal matrix whose trace decreases proportionally to the zoom magnitude when considering PTZ cameras. Note that the camera orientation $R_{W2B,i}$ in (2) is reported as a time-varying quantity since the provided observation model is valid both for the static and dynamic visual sensors.

### 2.5. VSN Modeling

Motivated by the intent of proposing a distributed solution, we assumed that any *i*-th visual sensor, $i\in \{1\ldots n_{C}\}$, composing the given VSN can communicate with the set of cameras placed in the same partition and in the adjacent ones. Formally, defining $C_{P_{k}}^{S}$ and $C_{P_{k}}^{D}$ as the sets of static and dynamic visual sensors located in the *k*-th partition, respectively, we have that all the devices placed in $P_{k}$ constitute the set $C_{P_{k}}=C_{P_{k}}^{S}⊕C_{P_{k}}^{D}$, $k\in \{1\ldots n_{P}\}$. Then, assuming that $C_{i}\in C_{P_{k}}$, we have that the cameras interacting with the *i*-th one at time *t* correspond to the set $C_{i}\left(t\right)\subseteq C_{P_{k}}\cup C_{P_{\kappa }}$, $P_{k}$ and $P_{\kappa }$ being adjacent partitions, $k,\kappa \in \{1\ldots n_{P}\}$, k≠κ. Note that we implicitly made the assumption that all cameras in the network are aware of the partition wherein they are located and also of the related handout zones.

**Figure 1:**
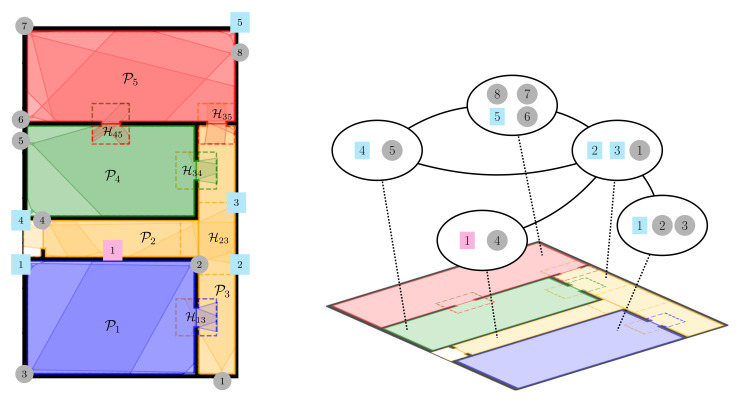
Environment example: colors distinguish the physical environment partitions; dashed lines identify the handout zones among virtual environment partitions; cameras are indicated with round and square markers.

Figure 1 aims at clarifying the introduced communication setup through a toy example fulfilling all the assumptions of the scenario taken into account in this work. In the following, the 3D simulation environment is shown using its projection on the 2D floor. One can, indeed, observe that the reported example envisages a structured environment having a single access point, composed of $n_{R}=4$ rooms (corresponding to the blue, red, green, and orange areas) and virtually divided into $n_{P}=5$ partitions connected by $n_{H}=5$ handout zones (dashed portions). We emphasize that the partitions $P_{2}$ and $P_{3}$ jointly cover the area associated with the orange room. The VSN is made up of $n_{SHR}=1$ high-resolution fixed camera (represented by the magenta square) monitoring the unique access point, $n_{SWA}=5$ wide-angle static visual sensors placed in the environment in order to guarantee the maximum area coverage (identified by the cyan squares), and $n_{D}=8$ PTZ cameras located with the purpose of entailing the network tracking capabilities (denoted by the gray circles). We point out that the FOV of the visual sensor $C_{2}^{SWA}$ can cover a portion of both the partitions $P_{2}$ and $P_{3}$; in addition, we remark that all the handout zones are potentially monitored by at least a dynamic visual sensor. On the right panel of Figure 1, we highlight the devices’ interaction in terms of information exchange: as illustrated, the cameras physically placed in the same partition can communicate among themselves, and these can also share data with the visual sensors located in the adjacent partitions. To conclude, we emphasize that the communication graph is imposed by the considered VSN structure. Similar graph-based descriptions, but with different connection roles among nodes were employed in [28,29] to characterize the environment structure.

## 3. Real-Time Surveillance and Multi-Target Tracking

Accounting for the scenario described in the previous section, we present here a distributed strategy aiming at ensuring the efficient real-time surveillance of the considered environment and the tracking of the $n_{T}$ targets, by means of the given VSN.

The designed procedure involves three principal actions:

- The *targets’ detection*, executed by both the static and dynamic visual sensors with the intent of extracting information regarding the presence of one or more targets;
- The *targets’ position estimation and prediction* performed by all the fixed and PTZ cameras having detected one or more targets, mainly to identify the devices involved in the tracking task in the near future;
- The *PTZ parameter selection*, carried out by all the dynamic visual sensors that are already or soon engaged in the tracking task, with the purpose of optimizing the real-time performance.

We specify that the first and second actions were performed at a frequency of 1/T, while the PTZ parameters were optimized every ℓ≥1 steps of duration *T*, namely at a slower frequency of 1/(ℓT) with respect to the previous ones. The parameter *ℓ* was selected in order to respect the computational limits of the system while guaranteeing its promptness in reacting to tracking requirements.

In the rest of the section, the attention is focused on the outlined methods for the estimation and prediction of the targets’ position and for the determination of the more suited parameters for the PTZ cameras. Conversely, we do not explicitly account for the targets’ detection, assuming that this action is accurately performed by resting upon one of the existing and well-proven techniques. On the other hand, we remark that the designed solution permits the computations’ parallelization. In detail, observing that both the targets’ detection and the PTZ parameter selection require a high computational burden, these two operations can be concurrently executed by distributing the workload between two computing cores, if possible. Indeed, the optimization process depends only on the information gathered at every *ℓ*-th step about the predicted target state.

### 3.1. Targets’ Position Estimation and Prediction

To efficiently fulfill the tracking task, it is well known that a fundamental step consists of the accurate estimation of the current position of the targets and also of their future trajectory. In the proposed strategy, we address this issue by suitably extending the distributed consensus-based EKF approach presented in [16] to the case of targets moving in 3D space (rather than 2D).

To better clarify the adopted approach, summarized in Algorithm 1, we focus on a generic *j*-th target, $j\in \{1\ldots n_{T}\}$, assuming that this is detected by a set $C_{T_{j}}\left(t\right)\subset C$ of cameras in the network. Observe that, according to (2), any *i*-th device in the aforementioned set can retrieve the projection $z_{ij}\left(t\right)$ of the target position onto its image plane jointly with the corresponding covariance $V_{i}$ (Line 2). This allows then computing the quantities $r_{ij}$ and $U_{ij}$ introduced in [16] (Lines 4–5). These are subsequently communicated to the devices set ${\bar{C}}_{i}\left(t\right)\subseteq C$ distinguishing between the following situations (lines 6–8).

     Assuming that $C_{i}\in C_{P_{k}}$, $k\in \{1\ldots n_{P}\}$, we have that:

- If the target is in $P_{k}\backslash H_{k,\kappa }$, $\forall H_{k,\kappa }\in H$, then the *i*-th device communicates with all the other cameras in the same partition, namely ${\bar{C}}_{i}\left(t\right)=C_{P_{k}}$;
- If the target is in $H_{k,\kappa }^{k}$ for any $\kappa \in \{1\ldots n_{H}\}$, then the *i*-th device communicates with all the cameras in the same and in the adjacent partition $P_{\kappa }$, i.e., ${\bar{C}}_{i}\left(t\right)=C_{P_{k}}⊕C_{P_{\kappa }}$.

**Algorithm 1**Distributed Consensus-based EKF1:**for** any detected target $T_{j}$ **do**2:   compute $z_{ij}\left(t\right)$ as in (2) and the corresponding $V_{i}$3:   compute $H_{ij}\left(t\right)\in R^{2\times 6}$ as $H_{ij}\left(t\right)=\nabla_{{\bar{x}}_{ij}\left(t\right)}h\left(R_{W2B,i}\left(t\right)\left(\left[\begin{array}{cc} I_{3\times 3} & 0_{3\times 3} \end{array}\right]{\bar{x}}_{ij}\left(t\right)\right)-t_{W,i})\right)$4:   compute $r_{ij}\left(t\right)=H_{ij}^{⊤}\left(t\right)V_{i}^{-1}z_{ij}\left(t\right)-H_{ij}^{⊤}\left(t\right)V_{i}^{-1}h_{i}\left({\bar{x}}_{ij}\left(t\right)\right)$ as in [16]5:   compute $U_{ij}\left(t\right)=H_{ij}^{⊤}\left(t\right)V_{i}^{-1}H_{ij}\left(t\right)$ as in [16]6:   *data exchange*7:   transmit $m_{ij}\left(t\right)=\left(r_{ij}\left(t\right),U_{ij}\left(t\right),{\bar{x}}_{ij}\left(t\right)\right)$ to any $C_{\iota }\in {\bar{C}}_{i}\left(t\right)$8:   receive $m_{\iota j}\left(t\right)=\left(r_{\iota j}\left(t\right),U_{\iota j}\left(t\right),{\bar{x}}_{\iota j}\left(t\right)\right)$ from any $C_{\iota }\in C_{T_{j}}\left(t\right)$9:   *information fusion*10:  compute $y_{ij}\left(t\right)=\sum_{C_{\iota }\in C_{i}\left(t\right)}r_{\iota j}\left(t\right)$11:  compute $S_{ij}\left(t\right)=\sum_{C_{\iota }\in C_{i}\left(t\right)}U_{\iota j}\left(t\right)$12:  *EKF* - *a posteriori estimation*13:  compute $M_{ij}\left(t\right)={\left(P_{ij}^{-1}\left(t\right)+S_{ij}\left(t\right)\right)}^{-1}$ (error covarince matrix)14:  compute ${\hat{x}}_{ij}\left(t\right)={\bar{x}}_{ij}\left(t\right)\;+\;M_{ij}\left(t\right)y_{ij}\left(t\right)\;+\;(∥M_{ij}\left(t\right){∥\;+\;1)}^{-1}\;M_{ij}\left(t\right)\sum_{C_{\iota }\in C_{T_{j}}\left(t\right)}\left({\bar{x}}_{\iota j}\left(t\right)-{\bar{x}}_{ij}\left(t\right)\right)$ (target state estimate)15:  *EKF* - *a priori estimation*16:  update $P_{ij}\left(t\right)=A_{j}\;M_{ij}\left(t\right)\;A_{j}^{T}+W_{j}$ (error covariance matrix)17:  update ${\bar{x}}_{ij}\left(t\right)=A_{j}\;{\hat{x}}_{ij}\left(t\right)$ (target state estimate)18:**end for**

The exchanged data are required by all the cameras in $C_{T_{j}}\left(t\right)$ to initialize and/or update an EKF needed to retrieve a suitable estimation ${\bar{x}}_{ij}\left(t\right)\in R^{6}$ of the *j*-th target state at time *t* (Lines 12–17). Note that the filter initialization can be performed exploiting either the received information or the environment knowledge, as, for instance, the size and position of the rooms’ access points. It is straightforward that the accuracy of such an estimation is affected by the number of cameras in $C_{T_{j}}\left(t\right)$. Moreover, it is possible to prove that, relying on the consensus approach, it holds that ${\bar{x}}_{ij}\left(t\right)={\bar{x}}_{j}\left(t\right)$ for any $C_{i}\in C_{T_{j}}\left(t\right)$, namely the target state estimation converges to the same value for all the devices detecting $T_{j}$. For this reason, hereafter, we drop out the dependence on the *i*-th camera when referring to the EKF target state estimation.

Our solution entails the exploitation of the computed target state estimation to determine (even roughly) a prediction of its evolution after ℓ>1 time steps. Indeed, exploiting the target dynamics (1), we obtain:

(3) $${\bar{x}}_{j}^{\ell }\left(t\right)=\left\{\begin{array}{cc} {\bar{x}}_{j}\left(t\right) & \;\mathrm{if}\;{\bar{p}}_{j}^{\ell }\left(t\right)\notin E\;\mathrm{or}\;\left({\bar{p}}_{j}\left(t\right)\notin H_{k\kappa }\;\&\;{\bar{p}}_{j}^{\ell }\left(t\right)\in P_{\kappa }\right)\\ {\bar{x}}_{j}\left(t+\ell \right)=A^{\ell }\;{\hat{x}}_{j}\left(t\right) & \;\mathrm{otherwise} \end{array}\right.,$$

where ${\bar{x}}_{j}^{\ell }\left(t\right)={\left[\begin{array}{c} {\bar{p}}_{j}^{\ell }\left(t\right)\;{\dot{\bar{p}}}_{j}^{\ell }\left(t\right) \end{array}\right]}^{⊤}\in R^{6}$ is the *ℓ*-steps ahead *j*-th target state prediction. Note that if the predicted target position ${\bar{p}}_{j}^{\ell }\left(t\right)\in R^{3}$ exits from the surveilled environment or if it changes partition without being in the corresponding handout zone, then the prediction is considered not valid and is substituted by the actual estimated position.

To conclude, we point out that the tracking performance is affected by the selected sampling time. Indeed, small values of *T* might imply extremely high computational burden, whereas large values of *T* might compromise the system promptness in the case of fast-moving targets. A good choice is to select the sampling time taking into account the average speed of the targets.

### 3.2. PTZ Parameter Selection

One of the most original aspects of the proposed surveillance and tracking solution rests upon the use of a heterogeneous VSN, through a smart exploitation of the adjustable parameters of the PTZ cameras. Hereafter, we illustrate the PTZ parameter selection procedure designed to determine both the orientation and the zoom value of the dynamic visual sensors in the network, with the purpose of improving the tracking capability of the whole camera group. In detail, inspired by [16], we tackled the selection of the PTZ cameras’ parameters through the iterative solution of a suitable maximization problem. Clearly, it is convenient to consider only a finite discrete number of PTZ parameters values since small changes do not yield relevant differences in the cameras’ FOV.

#### 3.2.1. PTZ Parameter Selection Procedure

To provide a clearer explanation, we first focus on the generic single *j*-th target, $j\in \{1\ldots n_{T}\}$. Based on the computed prediction (3) and exploiting the information on the network topology, it is possible to identify the set of both fixed and PTZ cameras that could potentially detect the considered target at the following *ℓ*-th time step. We indicate such a set as $C_{T_{j}}^{\ell }\left(t\right)=C_{T_{j}}^{\ell ,S}\left(t\right)⊕C_{T_{j}}^{\ell ,D}\left(t\right)$, distinguishing between the static and dynamic visual sensors subsets. In particular, we specify that if the target predicted position ${\bar{p}}_{j}^{\ell }\left(t\right)$ is in $P_{k}\backslash H_{k,\kappa }$, $\forall H_{k,\kappa }\in H$, $k,\kappa \in \{1\ldots n_{P}\},k\neq \kappa$, then the set $C_{T_{j}}^{\ell }\left(t\right)$ includes only cameras placed in the partition $P_{k}$. Instead, if after *ℓ* time steps, the target is estimated to be in $H_{k,\kappa }^{k}$, then the set $C_{T_{j}}^{\ell }\left(t\right)$ contains all visual sensors located in $P_{k}$ and only the static ones of $P_{\kappa }$. Formally, in the former scenario, we have that $C_{T_{j}}^{\ell }\left(t\right)\subseteq C_{P_{k}}$, while in the latter one, $C_{T_{j}}^{\ell }\left(t\right)\subseteq C_{P_{k}}\cup C_{P_{\kappa }}^{S}$.

The PTZ parameter selection process initially requires the communication among all the cameras in $C_{T_{j}}^{\ell }\left(t\right)$. The involved devices share information about their position, orientation, as well as zoom value in the case of PTZ cameras. Subsequently, all the PTZ cameras in $C_{T_{j}}^{\ell ,D}\left(t\right)$ compute a certain *utility function* depending on the received information. Then, for a fixed number m≥1 of consecutive iterations, a single dynamic visual sensor at a time, randomly selected from a uniform distribution over the set $C_{T_{j}}^{\ell ,D}\left(t\right)$, computes the optimal values for its PTZ parameters (maximizing the utility function) and broadcasts this information to the other cameras in $C_{T_{j}}^{\ell ,D}\left(t\right)$, which correspondingly update their utility function. The whole iterative procedure can be interpreted as a *negotiation phase*.

For sufficiently large values of *m*, such a negotiation phase allows the selection process to converge at least towards a local maximum. In particular, as proven in [16], the convergence is ensured by the game theory results on the Nash equilibrium. When both the number of cameras in a partition and the number of PTZ parameters to select are high, it could be advantageous to rely on a stochastic method for the PTZ parameter selection (for example, in [16], at each negotiation step, a softmax function and a temperature variable were used to generate a probability distribution over the utilities of the selected camera available configurations). This allows avoiding the local maxima, but turns out to be computational demanding. On the contrary, when both the number of visual sensors and the number of PTZ parameters to select is low (and therefore, the risk of incurring a local maximum is low) or when a sub-optimal solution is accepted to the benefit of a faster convergence, then a greedy choice method could be preferred.

Note also that, for the PTZ parameter selection, the dynamic visual sensors do not exchange data with the PTZ cameras placed in other partitions. This implies that the negotiation phase can be simultaneously performed in more than one partition, coping with the presence of multiple targets.

Finally, we emphasize that the selection process’s performance is conditioned by the value assigned to *ℓ*: the number of prediction time steps needs to be compatible with the value of *m* and the cameras’ computational and actuation time.

#### 3.2.2. Utility Function Definition

The determination of the PTZ parameters from any dynamic camera in $C_{T_{j}}^{\ell ,D}\left(t\right)$ relies on the evaluation of the aforementioned utility function. Such a function is computed tacking into account all the targets that the device is supposed to detect at the following *ℓ*-th time step. Denoting this targets set as ${}^{i}T\left(t\right)\subseteq T$, we define the *i*-th camera utility function as:

(4) $$f_{i}\left(\alpha_{i}\left(t\right),\beta_{i}\left(t\right),\zeta_{i}\left(t\right)\right)=\sum_{T_{j}\in^{i}T\left(t\right)}q_{j}f_{i}^{j}\left(\alpha_{i}\left(t\right),\beta_{i}\left(t\right),\zeta_{i}\left(t\right)\right)$$

where the triplet $\left(\alpha_{i}\left(t\right),\beta_{i}\left(t\right),\zeta_{i}\left(t\right)\right)$ summarizes the PTZ camera parameters, the scalar $q_{j}\geq 0$ constitutes the weight assigned to the *j*-th target in order to prioritize (or less) its tracking and the function $f_{i}^{j}\left(\alpha_{i}\left(t\right),\beta_{i}\left(t\right),\zeta_{i}\left(t\right)\right)$ depends only on the *j*-th target and on the position, orientation, and eventually, zoom value of the visual sensors belonging to the set $C_{T_{j}}^{\ell }\left(t\right)$. More in detail, $f_{i}^{j}\left(\alpha_{i}\left(t\right),\beta_{i}\left(t\right),\zeta_{i}\left(t\right)\right)$ is defined as the weighted sum of the terms deriving from the adoption of l≥0 different criteria, namely:

(5) $$f_{i}^{j}\left(\alpha_{i}\left(t\right),\beta_{i}\left(t\right),\zeta_{i}\left(t\right)\right)=\sum_{l}r_{l}g_{l}\left(\alpha_{i}\left(t\right),\beta_{i}\left(t\right),\zeta_{i}\left(t\right)\right)$$

with $r_{l}\geq 1$ and $g_{l}\left(\alpha_{i}\left(t\right),\beta_{i}\left(t\right),\zeta_{i}\left(t\right)\right)$ inferred as explained in the following.

In addition to those proposed in [16], in this work, we account for these criteria:

1. *Distance from the center*: This criterion implies the evaluation of the distance of the predicted position of the target from the center of the camera image plane. As a consequence, in this case, we have that:
  (6) $$\begin{array}{cc} \; & g_{1}\left(\alpha_{i}\left(t\right),\beta_{i}\left(t\right),\zeta_{i}\left(t\right)\right)=-\frac{1}{|C_{T_{j}}^{\ell }\left(t\right)|}\;\sum_{C_{\iota }\in C_{T_{j}}^{\ell }\left(t\right)}\chi_{\iota }p+ad\left(\alpha_{\iota }\left(t\right),\beta_{\iota }\left(t\right),\zeta_{\iota }\left(t\right)\right)\\ \; & \mathrm{with}\;d\left(\cdot \right)=∥\left[\begin{array}{cc} I_{2\times 2} & 0_{2\times 1} \end{array}\right]R_{W2B,\iota }\left(\alpha_{\iota }\left(t\right),\beta_{\iota }\left(t\right)\right)\left({\bar{x}}_{j}^{\ell }-t_{W,\iota }\right)∥. \end{array}$$
  In (6), the scalar a∈[0,1] permits weighting the importance given to the distance d(·), and it is set to one when the *i*-th camera can detect the target without modifying its PTZ parameters (Case *a*). On the other hand, the condition a<1 is in place when the *i*-th camera needs to modify its current orientation and/or zoom parameter in order to detect the target (Case *b*). Note that in this last scenario, a penalty p>0 is also assigned to the device thanks to the introduction of the indicator function $\chi_{\iota }$, which takes a value of one in correspondence to Case *b* and zero to Case *a*;
2. *View quality*: This criterion intends to favor zoomed frames up to a minimum FOV height $h_{min}>0$, which is measured on the plane orthogonal to the optical axis and intersecting the *j*-th target position. Hence, we account for:
  (7) $$\begin{array}{cc} \; & g_{2}\left(\alpha_{i}\left(t\right),\beta_{i}\left(t\right),\zeta_{i}\left(t\right)\right)=\sum_{C_{\iota }\in C_{T_{j}}^{\ell }\left(t\right)}\chi_{\iota }h_{\iota }\left(\alpha_{\iota }\left(t\right),\beta_{\iota }\left(t\right),\zeta_{\iota }\left(t\right)\right)\\ \; & \mathrm{with}\\ \; & h_{\iota }\left(\cdot \right)=\left\{\begin{array}{cc} {\left(\left[\begin{array}{c} 0\;0\;2 \end{array}\right]R_{W2B,\iota }\left(\alpha_{\iota }\left(t\right),\beta_{\iota }\left(t\right)\right)\left({\bar{x}}_{j}^{\ell }-t_{W,\iota }\right)\tan\left(\frac{1}{2}\beta_{\iota }\left(t\right)\right)\right)}^{-1} & \mathrm{if}\;h_{\iota }\left(\cdot \right)>h_{min}\\ 0 & \mathrm{otherwise}; \end{array}\right. \end{array}$$
3. *Number of cameras per target*: This criterion aims at assigning a penalty p>0 when the *j*-th target is estimated to be detected by less than $n_{min}$ cameras or more than $n_{max}$ cameras. Hence, we have that:
  (8) $$g_{3}\left(\alpha_{i}\left(t\right),\beta_{i}\left(t\right),\zeta_{i}\left(t\right)\right)=\left\{\begin{array}{cc} 0 & \mathrm{if}\;n_{min}\leq \left|C_{T_{j}}^{\ell }\left(t\right)\right|\leq n_{max}\\ -p & \mathrm{otherwise}; \end{array}\right.$$
4. *Minimum parameter adjustments*: According to this criterion, the minimum parameter adjustments with respect to the previous update step are advisable. Introducing the vector ${\left[\begin{array}{ccc} \alpha_{i}^{☆} & \beta_{i}^{☆} & \zeta_{i}^{☆} \end{array}\right]}^{⊤}$ stacking the PTZ parameters of the *i*-th camera obtained at the previous selection step, it follows that:
  (9) $$g_{3}\left(\alpha_{i}\left(t\right),\beta_{i}\left(t\right),\zeta_{i}\left(t\right)\right)=-∥{\left[\begin{array}{ccc} \alpha_{i} & \beta_{i} & \zeta_{i} \end{array}\right]}^{⊤}-{\left[\begin{array}{ccc} \alpha_{i}^{☆} & \beta_{i}^{☆} & \zeta_{i}^{☆} \end{array}\right]}^{⊤}∥.$$

To conclude, we remark that, when accounting for different rooms, only static visual sensors are allowed to communicate. For this reason, it is possible to perform the selection of the PTZ cameras in separate partitions simultaneously.

## 4. Application Scenario

We note that the framework proposed in this work allows coping with a wide range of different (and potentially conflicting) objectives. This is possible through a convenient choice of the utility function terms and of the corresponding weights. Nonetheless, in this section, the attention is focused on the description of a specific application scenario. This is motivated by the intent of investigating the performance of the designed solution. In detail, we considered an application case wherein the necessary tradeoff between the high-resolution and high-precision tracking requirements emerges.

We considered the indoor environment depicted in Figure 1. This is physically divided into $n_{R}=4$ portions, namely a corridor (where the main entrance to the surveilled area is located) and three rooms accessible from the corridor. However, $n_{P}=5$ virtual partitions were taken into account. Indeed, two virtual partitions are associated with the environment portion physically corresponding to the corridor; this choice was motivated by the space geometry and by the intent of preventing camera view occlusions.

Figure 1 reports also the assumed cameras’ placement in the environment. We highlight that the outlined framework allows verifying the simultaneous PTZ parameter selection for dynamic devices associated with different partitions.

### 4.1. VSN Insights

We emphasize that partitions $P_{1},P_{2}$, and $P_{3}$ are populated by the minimum number of visual sensors to guarantee multi-target tracking; partition $P_{4}$ is surveilled only by a (wide-angle) static and a dynamic device; four cameras are located in correspondence to partition $P_{5}$. Note that $P_{4}$ and $P_{5}$ represent the most critical and the most favorable situations in the considered framework, respectively.

**Figure 2:**
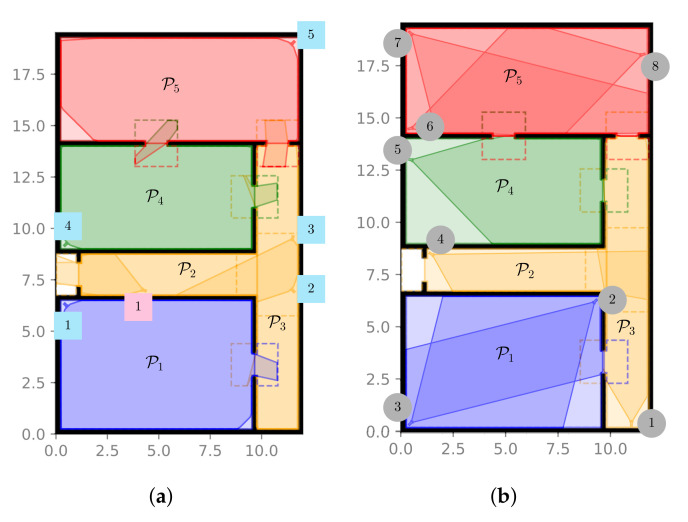
Simulation framework: cameras’ position. (**a**) Fixed cameras’ position. (**b**) PTZ cameras’ position.

More in detail, one can observe that, as highlighted in Figure 2a, the static visual sensors were placed in order to guarantee the monitoring of the whole environment. Nonetheless, different features were assumed for these cameras, as reported in Table 2. Observe that the high-resolution device aimed at monitoring the environment access point is characterized by a limited FOV. Concerning the PTZ cameras, instead, these are supposed to be located so as to ensure that the volume of each partition can be approximately entirely covered by at least a couple of these devices, except for partition $P_{4}$, as depicted in Figure 2b. The considered PTZ cameras were not all identical and differ, as reported in Table 3, where the pan and tilt ranges identify the extreme achievable angles when moving with respect to the initial configuration. Note that the sensors placed in the corridor are characterized by a smaller pan range as compared to the ones in the other rooms, which can span a larger area.

In the simulation, the FOV of each camera is also characterized by a maximum distance at which a target can be seen. This value can be computed starting from each camera resolution, horizontal and vertical FOV angles and the minimum pixel density at which a target is considered to be detectable. Clearly, for dynamic cameras, this maximum distance changes depending on the zoom magnitude. The minimum density considered by us was 3 $\mathrm{pixel}\;\mathrm{per}\;{\mathrm{cm}}^{2}$ (ppcm).

Taking into account a maximum velocity of 4m/s for the targets, we assume that all the cameras composing the VSN acquire new data every T=50ms. In addition, for any *i*-th static sensor, $i\in \{1\ldots n_{S}\}$, we select the covariance matrix of its observation error in (2) as a diagonal matrix $V_{i}\left(t\right)=10^{-3}I_{3\times 3}$. In doing this, when projecting the target position on the camera image plane, we have that the maximum error is approximately 9.5 cm for a target at a distance of 1 m. Conversely, for any ι-th PTZ camera, $\iota \in \{1\ldots n_{D}\}$, the covariance matrix depends on the value of its zoom parameter as $V_{\iota }\left(t\right)=\left(10^{-3}/\zeta_{\iota }\left(t\right)I_{3\times 3}\right)$. Note that we suppose that the zoom parameter can vary in the range [1,3] with unitary step for all the dynamic devices (Table 3). The pan and tilt angles, instead, can be updated in different ranges for the various cameras, although the update step is fixed to 7.5 degrees for all the devices. We also assume that such angular movements are achieved in 0.5 s per step: this constitutes an arbitrary choice, even though, in the following, it is shown that reasonably longer movement time do not affect considerably the tracking performance.

### 4.2. PTZ Parameter Selection Insights

In the following, the PTZ parameter selection is performed by relying only on two of the criteria presented in Section 3.2, namely the *distance from the center* and the *quality view* criterion. This choice was motivated by the results of a preliminary comparison of all the proposed criteria, jointly with those described in [16]: the two selected ones constitute the best tradeoff in terms of both effectiveness and computational burden. We remark that the *tracking* criterion introduced in [16] and the outlined *distance from the center* criterion serve the same purpose. Nonetheless, the former requires complex computations to minimize the covariance matrix of the target state estimate, while the latter ensures good tracking performance just by trying to keep the targets as centered as possible in the image plane, only involving the computation of the distance from the optical axis through a norm. As regards the *distance from the center* criterion, the penalty term and the weight factor introduced in (6) were set to $p=10^{3}$ and a=0.5, respectively. As far as the *quality view* criterion is concerned, instead, the parameter $h_{min}$ in (7) was fixed to 1 m. We highlight that the purpose of the studied scenario was to obtain high-resolution shots of the targets while maintaining a good tracking performance for all of them. Since the *quality view* criterion favors zoomed framings of the targets and the *distance from the center criterion* improves the tracking precision, just by combining these two simple rules, it is possible to obtain the desired network behavior.

In light of the given premises, the utility function computed by any *i*-th PTZ camera, $i\in \{1\ldots n_{D}\}$, in correspondence to the generic *j*-th target, $j\in \{1\ldots n_{T}\}$, results in being:

(10) $$f_{i}^{j}\left(\alpha_{i}\left(t\right),\beta_{i}\left(t\right),\zeta_{i}\left(t\right)\right)=r_{1}\;g_{1}\left(\alpha_{i}\left(t\right),\beta_{i}\left(t\right),\zeta_{i}\left(t\right)\right)+r_{2}\;g_{2}\left(\alpha_{i}\left(t\right),\beta_{i}\left(t\right),\zeta_{i}\left(t\right)\right)$$

with $g_{1}\left(\alpha_{i}\left(t\right),\beta_{i}\left(t\right),\zeta_{i}\left(t\right)\right)$ and $g_{2}\left(\alpha_{i}\left(t\right),\beta_{i}\left(t\right),\zeta_{i}\left(t\right)\right)$ defined as in (6) and (7), respectively, and $r_{1}=r_{2}=1$, namely without prioritizing any of the two selected criteria. The utility function that the *i*-th PTZ camera is required to maximize in order to determine its next PTZ parameter is thus $f_{i}\left(\alpha_{i}\left(t\right),\beta_{i}\left(t\right),\zeta_{i}\left(t\right)\right)$ defined in (4). In particular, hereafter, we assume $q_{j}=1$ for any $j\in \{1\ldots n_{T}\}$.

We remark that the PTZ parameter selection can be simultaneously performed by devices corresponding to different partitions. Moreover, since in our simulation framework, at most three dynamic visual sensors are placed in each partition, for the reasons explained in Section 3.2.1, a greedy selection policy over the PTZ parameters was employed. Therefore, it is possible to use a relatively small number of negotiation steps, i.e., m=6. Observe that partitions $P_{1}$, $P_{2}$, and $P_{4}$ are all monitored by a single dynamic sensor: in these cases, the optimal PTZ parameters are directly determined and the negotiating process is not required. To conclude, we highlight that the computational time for parameter selection is proportional to the number of targets inside a partition. Observing also that the network tracking capabilities are strongly influenced by the PTZ parameter selection time, we made the following design choices as regards the heterogeneous network. First, the dynamic devices are supposed to be able to perform a discrete pan and/or tilt movement in 0.5s. In addition to this time, we need to consider also a small time interval during which the cameras stand still in order to acquire images without motion blur. We chose this interval to be of at least 0.5s, during which we also computed the new PTZ parameters for the dynamic cameras. It follows that, if the computational time exceeds this value, the mentioned time interval needs to be longer. As a consequence, it turns out to be convenient to predict the targets state at least 1s in the future. Having assumed T=0.05s, this leads to selecting ℓ≥20 prediction time steps, 10 steps of which are introduced to cover the camera movement time.

### 4.3. Targets’ Insights

In the designed simulation framework, we accounted for multiple possible targets moving in the environment according to (1). In detail, we selected the *j*-th target state noise covariance matrix, $j\in \{1\ldots n_{T}\}$, as:

(11) $$W=∥{\bar{p}}_{j}∥\left[\begin{array}{cc} 0.25I_{3\times 3} & 0_{3\times 3}\\ 0_{3\times 3} & 2.5I_{3\times 3} \end{array}\right]$$

where we indicate with $∥{\bar{p}}_{j}∥\geq 0$ the average target velocity expressed in m/s.

**Figure 3:**
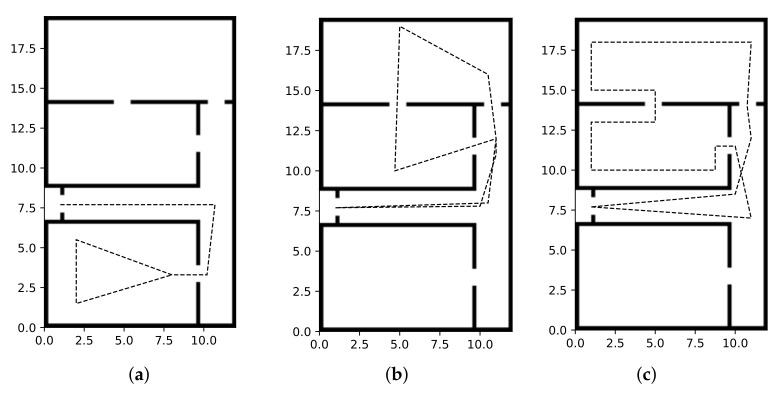
Possible target trajectories (without noise). (**a**) Trajectory T1, (**b**) Trajectory T2, (**c**) Trajectory T3.

In addition, we took into account three main possible trajectories for all the targets: these are reported in Figure 3, neglecting the hypothesis of additional noise. The path in Figure 3a, i.e., Trajectory T1, refers to a *non-elusive target* going through partitions $P_{1}$, $P_{2}$, and $P_{3}$, i.e., the environment portion characterized by a minimum number of cameras to properly ensure the target tracking. The trajectories in Figure 3b,c, namely T2 and T3, respectively, account for the behavior of a non-elusive and an elusive target, respectively, crossing partitions $P_{4}$ and $P_{5}$. We recall that these two partitions represent the most critical and favorable scenario in terms of cameras to guarantee the target tracking. In all three cases, targets were generally assumed to move at a constant speed of 1m/s, although variations up to 4m/s were also studied for the trajectory in Figure 3a. In addition, in the following, we considered scenarios wherein 4, 8, and 12 targets were simultaneously present in the environment. In these cases, the distance among them was reduced in order to have all of subjects concurrently present in $P_{1}$ at some point during the simulation.

## 5. Simulation Results

Accounting for the simulation framework outlined in the previous section, hereafter, we investigate the performance of the solution designed, by studying different scenarios.

### 5.1. Performance Evaluation Criteria

To do this, the following performance indexes were taken into account:

- The target state estimation error (*precision*) and the tracking confidence (*accuracy*). For any *j*-th target, $j\in \{1\ldots n_{T}\}$, at time *t*, the former is simply the difference between the true and the estimated state. The latter is computed from the elements on the main diagonal of the covariance matrix $P_{j}\left(t\right)$. Formally, we distinguish between the position estimation accuracy $\delta_{p}\left(t\right)=\sqrt{3(p_{1,1}\left(t\right)+p_{2,2}\left(t\right)+p_{3,3})}\in R$ and the velocity estimation accuracy $\delta_{v}\left(t\right)=\sqrt{3(p_{4,4}+p_{5,5}+p_{6,6})}\in R$. Note that the accuracy indexes $\delta_{p}\left(t\right)$ and $\delta_{v}\left(t\right)$ correspond to the 3σ C.I., where σ is the square root of the average variances of the position and velocity components of the target state, respectively;
- The (maximum, minimum, mean, and 75th percentile) resolution at which any target is observed. This is expressed in $\mathrm{pixel}\;\mathrm{per}\;{\mathrm{cm}}^{2}$ (ppcm) and computed evaluating the pixel density per $1\;m^{2}$ on the plane orthogonal to the optical axis and intersecting the estimated target position;
- The number of cameras detecting any target at each time step *T*;
- The time required for the PTZ parameter selection depending on the number of targets in the partition.

Note that only the last mentioned performance index involves a temporal quantity and, specifically, refers to the computational time employed in the PTZ parameter selection. This choice was motivated by the fact that the workload associated with all the other operations, as, e.g., the EKF target state estimation, is negligible with respect to the PTZ parameter selection process.

**Figure 4:**
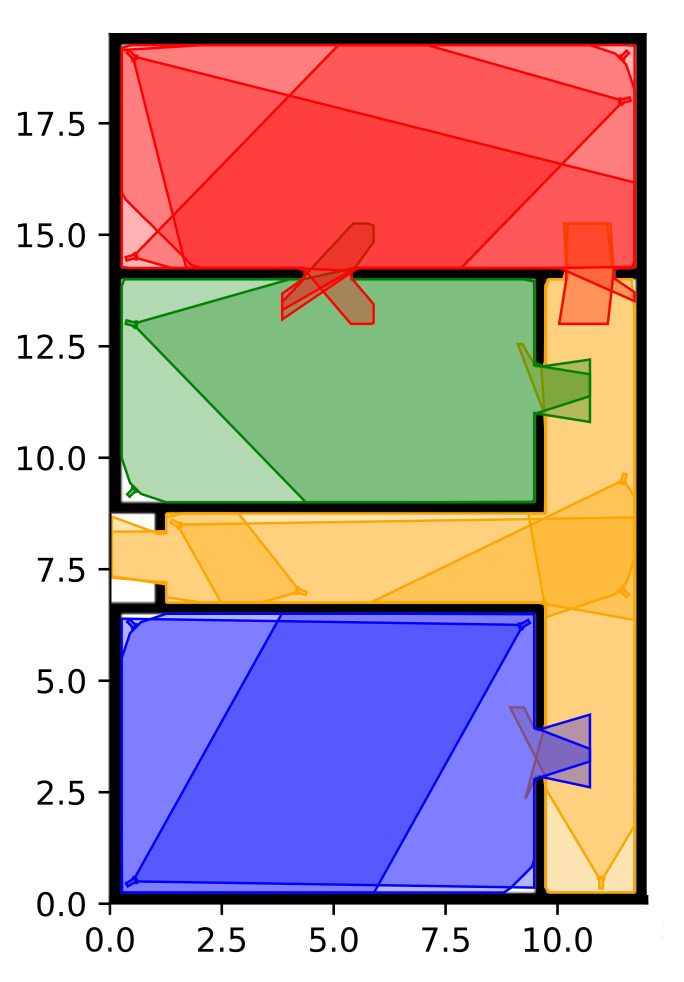
Static Simulation Framework (SSF).

Furthermore, to better highlight the advantages of heterogeneous VSNs including also PTZ cameras, we introduce the so-called *static simulation framework* (SSF). This differs from the simulation framework described in Section 4, hereafter termed the *dynamic simulation framework* (DSF), since we assumed substituting all the dynamic visual sensors with static devices. In particular, in the SSF, we fixed the orientation of the (substituted) cameras in order to have at least a couple of sensors monitoring the whole volume of each partition, except for partition $P_{4}$, as depicted in Figure 4.

Since we did not deal with occlusions, one can realize that in the SSF, the network tracking performance does not scale with the number of targets. For this reason, in the following, we investigate the performance of the SSF by accounting only for a single target moving in the environment. Nonetheless, the achieved results were then used as a benchmark for comparing the performance of the designed solution in the given DSF, specifically addressing also the multi-target case. All the simulations were run on a Windows laptop equipped with an Intel core i7-6700HQ.

To conclude, we emphasize that, to provide a fair evaluation of the designed solution performance, 10 independent trials were run for each testing scenario and the average of all the aforementioned metrics was computed.

### 5.2. Single Target

The first intent is to remark about the advantages deriving from the use of a heterogeneous VSN. In doing this, we focus on the network tracking capabilities both in the SSF and in the DSF, by considering a single target that follows the trajectories described in Section 4.3 with a constant speed equal to 1m/s.

First, note that in this case, the choice of ℓ=20 prediction steps (Section 4.2) is sufficient. Indeed, as shown in Table 4, the computational time required for the PTZ parameter selection in the case of a single target following the trajectory T3 (in Figure 3c) never exceeded 0.5s. We remark that the PTZ parameter selection procedure took into account not only the visible targets, but also the ones potentially visible in the near future. For this reason, the computation time turned out to be strongly affected also by the environment structure: a PTZ camera located in a room having a complex geometry in terms of walls and obstacles is penalized.

**Figure 5:**
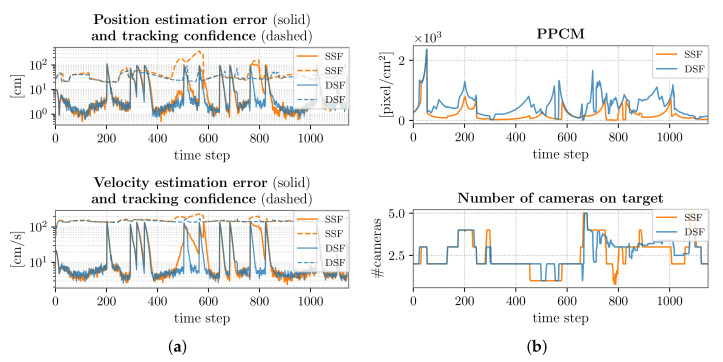
SSF vs. DSF performance comparison: 1 target following trajectory T3. (**a**) Position and velocity estimation error and tracking confidence. (**b**) PPCM and num. cameras on target evolution.

Focusing on the target tracking of trajectory T3 (the most challenging one for the camera network), in Figure 5, we report the performance indexes in correspondence to both the SSF and the DSF. However, we also summarize the performance in correspondence to all the target trajectories that are depicted in Figure 3 and Table 5.

**Figure 6:**
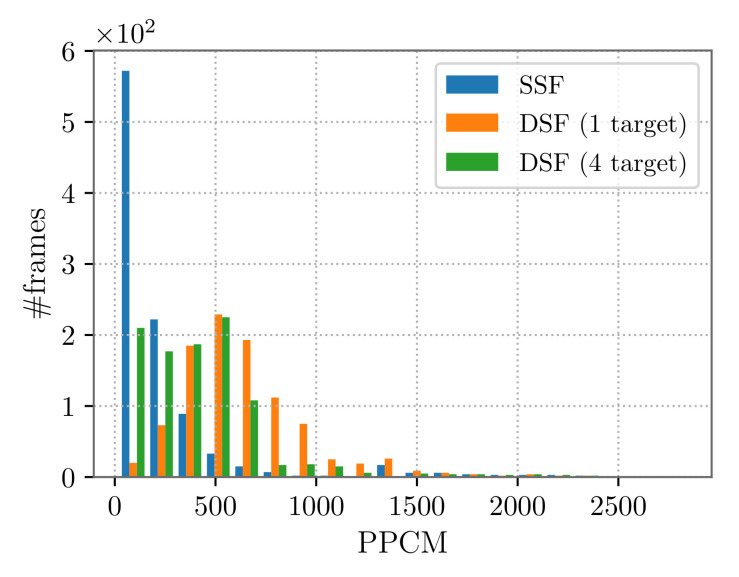
SSF vs. DSF frame distribution: 1 and 4 targets following trajectory T1.

It is possible to notice that the estimation error and tracking confidence, namely the position and velocity precision and accuracy, are comparable for all the trajectories, with a slight improvement when considering the DSF. This improvement can be explained by observing the mean number of cameras on the target. Indeed, accounting for Figure 5b, related to the case of a target following trajectory T3, we note that in several steps of the path, the number of cameras framing the target was larger for the DSF, as compared to the SSF. Moreover, the use of PTZ cameras allows considerably improving the resolution at which the target is seen (see Table 5 and Figure 5b). Observing also Figure 6, one can realize that the frame distribution in terms of ppcm is higher in DSF.

We observed that some spikes affected the trend of the estimation error reported in Figure 5a: this fact can be motivated by the changes of direction in the considered trajectory, approximated with instantaneous variations. However, the overall performance was not compromised by this behavior: the maximum value of the tracking precision and accuracy was, indeed, bigger than its 75th percentile. We emphasize that in real-world scenarios, this issue is less remarkable, since usually, changes of direction happen more smoothly; nonetheless, the proposed approach is useful to test the robustness of the network tracking capability.

In the elusive target scenario corresponding to trajectory T3 (Figure 5), we highlight that the target tries to exploit the blind areas of the VSN, and this turns out to be particularly problematic in partition $P_{4}$, where the visual sensors are not capable of ensuring the tracking over the entire environment portion. This fact can be noticed in Figure 5a: in correspondence to the part of the trajectory associated with partition $P_{4}$, namely between the 350th and the 650th time step, the accuracy and precision are compromised, especially when considering the SSF, since the target is framed by only a single camera for a long time (Figure 5b). In the DSF, the presence of dynamic devices allows partially counteracting this problem, with the limiting factor given by the movement capabilities of the PTZ cameras.

To conclude, to test the robustness inherited from the distributed approach, two scenarios were analyzed where cameras $C_{7}^{D}$ and $C_{8}^{D}$ were respectively considered as not working and a target was following trajectory T2. In both cases, thanks to the negotiation among the remaining cameras, the VSN was able to autonomously adapt to the new situation and to limit the loss of performance to a slight decrease in the quality of view performance (mean resolution values), as can be observed by comparing the data in Table 5 (DSF, T2) and Table 6.

### 5.3. Single Target vs. Four Targets

**Figure 7:**
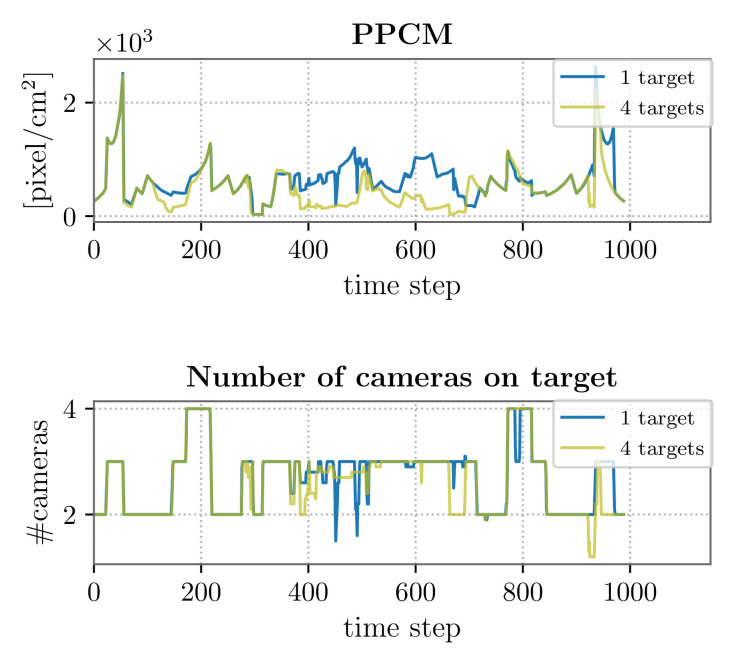
DSF performance: 1 and 4 targets following trajectory T1.

Accounting for the DSF, in Figure 7, we compare the single-target case discussed in the previous subsection with the case in which four targets move in the environment following trajectory T1 and thus crossing partitions $P_{1}$, $P_{2}$, and $P_{3}$. The intent, here, is to evaluate the heterogeneous VSN performance in a scenario wherein the number of cameras is the minimum to guarantee a successful target tracking over the entire area.

First of all, from Table 7, it is possible to notice that also with four targets, the computational time necessary to perform the PTZ parameter selection did not exceed 0.5s, thus the assumption on the target state prediction ℓ=20 is again valuable.

Based on the accuracy and precision values reported in Table 8, we point out that the increase in the number of targets did not affect the network tracking performance. However, when multiple targets were simultaneously present in the same partition, the PTZ cameras leaned toward the reduction of their zoom value in order to frame as many targets as possible, consequently compromising the (mean) resolution at which the targets were observed. This was due to the fact that the utility function (4) was designed so that all the dynamic devices tend to keep all targets at the maximum possible resolution, but also centered in their camera image plane. The described situation is more probable in larger partitions, as can be noticed from the ppcm trend in Figure 7: between the 400th and the 700th time step, we detected the highest discrepancy between the single- and multi-target case, and this corresponds to the part of the target trajectory in $P_{3}$. We also observed that the utility function maximization sometimes led the PTZ cameras to focus on a single target (thus, increasing their zoom value), rather than framing more targets, especially when these were already monitored by other visual sensors. This fact explains why the mean number of cameras on the targets was slightly lower in the DSF when accounting for four targets as compared both to the one-target case and to the SSF (see Table 8). In general, it is possible to balance the amount of zoomed PTZ configurations with a good tracking performance by tuning the weights of the target utility function.

To conclude, we highlight that Figure 6 and Table 5 and Table 8 show that, even when four targets are assumed to be present in the environment, the use of PTZ cameras brings a benefit in terms of target resolution. In fact, the average pixel density on the target was higher with respect to the one obtained in SSF.

### 5.4. Multiple Targets: Limit Behavior

Hereafter, we analyze how the average target resolution changes when the number of targets increases, and in particular when the use of PTZ cameras in the VSN does not provide any improvement with respect to the SSF. In doing this, we considered two scenarios involving 8 and 12 targets, respectively, ensuring their simultaneous passage through the partition $P_{1}$ at some time instant. It is straightforward that the different partitions have different tracking limits, depending on their topology and on the number of corresponding visual sensors. To provide a fair comparison, we considered trajectory T1 as in Section 5.3.

It turned out that the tracking limit in the DSF was 12 targets. Indeed, as can be observed comparing Table 5 and Table 9, the advantage given by the exploitation of a heterogeneous network in this case was minimal. As a consequence, we can conjecture that for a higher number of targets, the network performance might degenerate until the use of PTZ devices results in not being beneficial. This happened, for instance, in partition $P_{2}$ and $P_{3}$ in correspondence to a lower number of targets because of the presence of a single dynamic device. On the other hand, in partition $P_{1}$, the adoption of a heterogeneous VSN turned out to be advantageous due to the higher number of available PTZ cameras. For the 12-target case, thus, the DSF performance in terms of mean ppcm was the same as can be obtained by considering a network of static cameras, with higher resolution in critical points of the surveilled environment.

**Figure 8:**
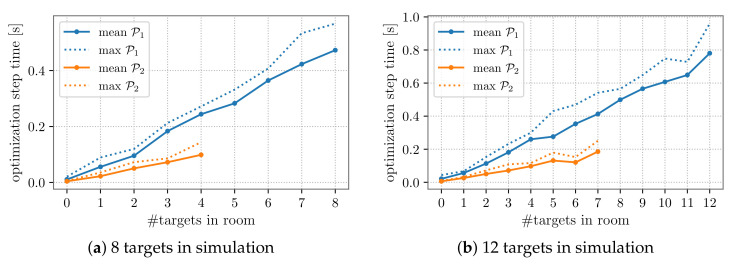
PTZ parameter selection computational time trend: results for partition $P_{3}$ are similar to those of partition $P_{2}$ and thus omitted. Note that the parameter selection process of PTZ cameras in $P_{2}$ depends on 4 over 8 targets (**a**) and 7 over 12 targets (**b**).

Moreover, Figure 8a,b highlights that the computational time grew approximately linearly with the number of targets in a partition and the trend slope changed according to the number of devices placed in the partition. One can also observe that in these cases, the computational time to select the PTZ parameters of all the dynamic cameras was higher than 0.5s (see Table 10 and Table 11). This implies that a longer time period needs to be taken into account for computations before the cameras’ movements. In particular, we considered intervals of 1s for the 8-target case and of 2s for the 12-target case, which correspond to ℓ=30 and ℓ=50 steps ahead prediction, respectively.

Finally, we noticed that, under the target velocity assumption of 1m/s, the target state estimation precision was not compromised (see Table 9), suggesting that the designed solution was capable of coping with situations involving a lower number of targets and a longer time for the camera movements.

### 5.5. Multiple Targets with Different Velocities

We investigate now the system performance in the presence of targets having different (increasing) velocities. Specifically, we assumed dealing with four targets moving at the constant speeds of 1m/s, 2m/s, and 4m/s. The last case is extreme for walking targets, but this was studied with the purpose of pushing the system performance and analyzing the provided solution robustness. Furthermore, in this scenario, the targets were supposed to follow trajectory T1.

**Figure 9:**
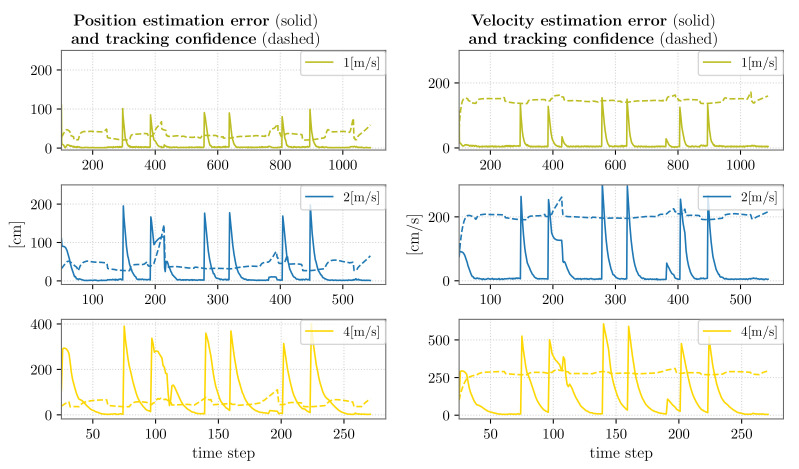
DSF tracking precision and accuracy: multiple targets having different velocities.

In Figure 9, we report the results of the comparison of the tracking precision and accuracy for the second of the four targets, while moving at the different velocities. We observed that, in all the cases, the distributed EKF generally allowed estimating the target position with a small error, except in correspondence to direction changes wherein the tracking error grew proportionally to the velocity. In particular, we note that for higher velocities, a longer time was required to correct the estimates. These considerations are confirmed by the results in Table 12. Nonetheless, we also remark that abrupt changes in direction are unlikely in a real case scenario, especially in the case of high velocities.

As concerns the resolution at which the targets were observed, from Table 12, one can observe that generally, the mean ppcm value was lower for higher target velocities. In these cases, in fact, less zoomed PTZ configurations are preferable since the movement that any camera can accomplish in one time step is not sufficient to follow a relatively close target that is moving fast.

Finally, we emphasize that, as the target velocity increases, the mean number of cameras on the target decreases. Indeed, in correspondence to faster targets, it is more likely for the PTZ cameras to temporarily lose the target. In these cases, the target state estimate only relies on the measurements of the static devices. In the worst case, for a brief time interval, the target is viewed by a single fixed camera; therefore, its state estimates can become less accurate until a PTZ camera frames it again.

### 5.6. Multiple-Target Real-World Scenario

To conclude the assessment of the proposed solution, we accounted for a real-world scenario wherein several targets access the surveilled environment with small time intervals between each other. They are all supposed to move at 1m/s, following different trajectories in order to cover the whole environment and to explore also the blind areas for the VSN. In this real-world scenario, the targets follow more natural paths with respect to the ones considered in the previous cases; the result is a more random distribution of the tracked subjects over the simulation area. We observed that the PTZ parameter selection computational time here was less than 1s for the partitions of the corridor and less than 0.5 s for the other partitions; hence, the prediction time steps considered were ℓ=30 in correspondence to partitions $P_{2}$ and $P_{3}$ and ℓ=20 for the other ones.

In this scenario also, particularly challenging from a surveillance point of view, the designed solution based on the exploitation of a heterogeneous VSN resulted in being more effective with respect to the SSF, especially in terms of the resolution at which targets were seen. This fact is confirmed when comparing the SSF and DSF performances reported in Table 13: the ppcm index almost doubled in correspondence to any partition, with the exception of $P_{2}$ and $P_{3}$, where the improvement was slightly less due to the constrained topology of the corridor.

**Figure 10:**
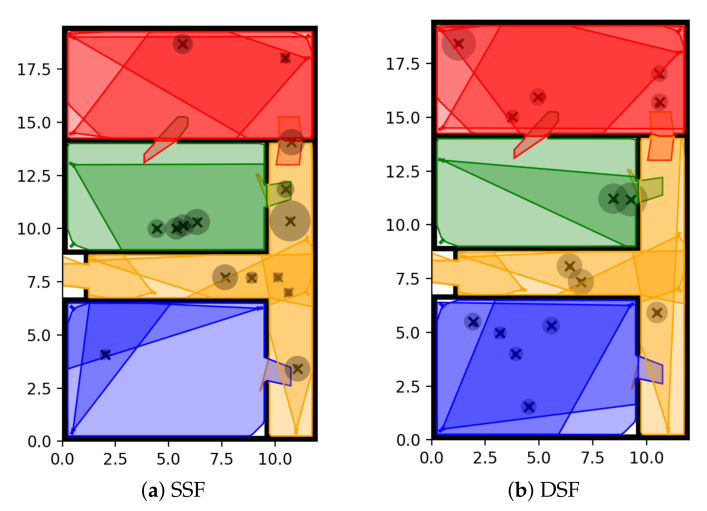
Snapshots of a real-world scenario in the DSF: (**a**) 5 targets are present in $P_{4}$; (**b**) 5 targets are present in both $P_{3}$ and $P_{5}$. The true position of the target is represented with a dot, its estimated position with a cross, and the confidence interval with a grey circle around the estimated position.

Furthermore, we remark that when multiple targets are present in a single partition, the number of PTZ cameras on a specific target tends to diminish. This behavior can be explained by considering that, based on the maximization of its utility function, for some dynamic device, it may to be more convenient to set its PTZ parameters in order to focus on a specific target, obtain some high-resolution shots instead of framing a larger area. The most challenging situation occurs when the maximum number of targets occupies a single partition and, in particular, these are spread over the entire partition area, e.g., when eight targets are present in partitions $P_{2}$ and $P_{3}$ and/or when five targets are present in partitions $P_{1}$, $P_{4}$, and $P_{5}$, as shown in Figure 10. In this case, it turns out to be preferable to not focus on a single target for a long period, since the risk of loosing the others exists. The results reported in Table 14 highlight how, besides the difficult situation, the solution proposed in this work was able to improve considerably the resolution at which targets were seen as compared to the traditional VSN characterizing the SSF.

## 6. Discussion

Accounting for the critical aspects of the proposed solution highlighted in the previous sections, we discuss here some possible ruses to improve the system performance.

First of all, we observed that the parameter selection computational time linearly depends on the number of targets, while the mean resolution at which the target is seen results in being inversely proportional. To address the first issue, it could be convenient to consider an adaptive rate for the PTZ parameter selection, namely to change the interval between consecutive selection procedures. To do so, it is sufficient to estimate the duration of the selection procedures’ process depending on the number of targets in a partition and therefore to select an adequate number of steps *ℓ* for the prediction of the target state. Indeed, when the partition is populated by a small number of targets, then a high PTZ parameter update rate can be adopted, thus improving the network performance. Note that the value of *ℓ* can be different for each partition, since the selection procedures can be carried out independently. Moreover, when the number of targets in the surveilled environment is such that the mean resolution obtained on them with a heterogeneous VSN is comparable to the one obtained with a static network, it could be useful to temporarily switch to a predefined configuration for the PTZ cameras that allows covering the entire area well, without trying to maximize the utility function.

Furthermore, we remark that, as the targets’ velocity increases, less zoomed configurations are preferred to reduce the possibility of losing the target. An improvement rests upon the introduction of an adaptive zoom based on the estimated velocity of the target, namely a procedure entailing the reduction of the maximum zoom magnitude as the velocity increases, allowing reducing the number of parameter value possibilities considered in the selection process.

Another crucial aspect is the management of the target direction changes, constituting the major source of error in the tracking process. To face this issue, it is possible to identify specific zones in which these situations are more likely to occur, such as the corners of a room or of the corridor, as well as the intersections between different rooms. When the targets are in these zones, it could be useful to consider higher values for the variance of the noise $w_{j}\left(t\right)$. This would allow dealing better with the uncertainty related to the changes of direction, which in these areas are bound to happen with a very high probability. Another more heuristic approach to this problem could be to consider only configurations with a wide FOV for cameras framing targets crossing these critical zones that would therefore be able to cover all the potential targets movements.

Finally, we emphasize that target occlusions represent still an open challenge in camera network design and in the proposed solution. It could be possible to select the PTZ cameras’ parameters according to the potential detectable obstacles present in the environment. Along this line, it would be sufficient to weight the contribution of each camera to a given target utility function according to the occlusion information. In other words, once a visual sensor realizes that a target is occluded by using its visual information jointly with the target state estimation, it could lower its contribution to the utility function with respect to the occluded target. In this way, the PTZ parameters of such a camera will be set by the algorithm to focus only on the targets that are visible from its point of view.

One further possible refinement could be to optimize the number, position, and type of cameras located inside the surveilled environment, for example by using the solutions proposed in [7,8,9,27]. This would allow starting from more advantageous PTZ parameter configurations as concerns the utility function maximization.

## 7. Conclusions

In this work, we proposed an original real-time surveillance and multi-target solution for an indoor environment, based on the exploitation of a heterogeneous VSN composed of both fixed and PTZ cameras. The environment topology was exploited to support the implementation of a distributed approach and thus obtain a robust, flexible, and scalable network. Indeed, the outlined structure allows separating the surveilled area into multiple independent partitions that can be handled in parallel.

The described surveillance solution consists of two main parts: a distributed EKF and a PTZ parameter selection algorithm that is based on a game theoretic approach. The former aims at estimating and predicting the state of the targets moving in the surveilled area. The latter, instead, given the predicted targets’ states, tries to maximize a utility function with the aim of finding the best configuration for the dynamic cameras in the VSN. Such a framework allows realizing a wide range of different and potentially conflicting objectives by simply choosing the proper utility function terms and weights.

In the simulation part, we evaluated the performance of the designed solution considering a specific case where the objective was to obtain a tradeoff between high-resolution views and good tracking ability. Multiple scenarios were investigated, by considering different numbers of targets following multiple trajectories at different velocities. The results confirmed the effectiveness of the solution in obtaining the desired tradeoff. In addition, it is possible to establish a linear relationships between the number of targets in a partition and the computational times. Finally, the threshold (in terms of target number) at which it is more convenient to temporarily switch to a static camera network configuration was also individuated.

## Figures and Tables

**Table 1:** Principal sets and main assumptions considered in this work.

Sets		Assumptions
environment access points	$A=\{A_{1}\ldots A_{n_{A}}\}$	$n_{A}\geq 1$
physical environment partitions	$R=\{R_{1}\ldots R_{n_{R}}\}$	$n_{R}\geq 1$
virtual environment partitions	$P=\{P_{1}\ldots P_{n_{P}}\}$	$n_{P}\geq n_{R}$
handout zones	$H=\left\{H_{1}\ldots H_{n_{H}}\right\}=\left\{H_{k\kappa }=H_{k\kappa }^{k}⊕H_{k\kappa }^{\kappa },\right\}$	$n_{H}\geq n_{P}$
static high-resolution visual sensors	$C^{SHR}=\left\{C_{1}^{SHR}\ldots C_{n_{SHR}}^{SHR}\right\}$	$n_{SHR}\geq n_{A}$
static wide-angle visual sensors	$C^{SWA}=\left\{C_{1}^{SWA}\ldots C_{n_{SWA}}^{SWA}\right\}$	$n_{SWA}\geq n_{P}$
static visual sensors	$C^{S}=\left\{C_{1}^{S}\ldots C_{n_{S}}^{S}\right\}=C^{SWA}⊕C^{SHR}$	$n_{S}=n_{SHR}+n_{SWA}\geq n_{P}+n_{A}$
dynamic visual sensors	$C^{D}=\left\{C_{1}^{D}\ldots C_{n_{D}}^{D}\right\}$	$n_{D}\geq n_{P}$
visual sensors	$C=\left\{C_{1}\ldots C_{n_{C}}\right\}=C^{D}⊕C^{S}$	$n_{C}=n_{D}+n_{S}\geq 2n_{P}+n_{A}$
targets	$T=\{T_{1}\ldots T_{n_{T}}\}$	$n_{T}\geq 1$

**Table 2:** Simulation framework: fixed cameras’ features.

Camera	FOV (deg)		Resolution (pixel/deg)
	Horiz.	Vert.	
$C_{1}^{SHR}$	60	40	100
$C_{1}^{SWA}$	120	80	50
$C_{2}^{SWA}$	120	80	50
$C_{3}^{SWA}$	90	60	50
$C_{4}^{SWA}$	120	80	50
$C_{5}^{SWA}$	120	80	50

**Table 3:** Simulation framework: PTZ cameras’ features.

Camera	FOV [deg]		Resolution (pixel/deg)	Tilt Range (deg)	Pan Range (deg)	Pan/Tilt Step (deg)	Zoom Range (deg)	Zoom Step (deg)
	Horiz.	Vert.						
$C_{1}^{D}$	60	40	50	±15	±15	7.5	1÷3	1
$C_{2}^{D}$	60	40	50	±15	±45	7.5	1÷3	1
$C_{3}^{D}$	60	40	50	±15	±45	7.5	1÷3	1
$C_{4}^{D}$	60	40	50	±15	±30	7.5	1÷3	1
$C_{5}^{D}$	60	40	50	±15	±30	7.5	1÷3	1
$C_{6}^{D}$	60	40	50	±15	±15	7.5	1÷3	1
$C_{7}^{D}$	60	40	50	±15	±30	7.5	1÷3	1
$C_{8}^{D}$	60	40	50	±15	±30	7.5	1÷3	1

**Table 4:** PTZ parameter selection computational time: 1 target following trajectory T3.

Case Study	Computational Time (s)	
1 target	mean	0.043
	std	0.036
	max	0.136

**Table 5:** SSF vs. DSF performance comparison: 1 target following trajectories T1, T2, and T3.

Case Study		Position Precision (cm)	Position Accuracy (cm)	Velocity Precision (cm/s)	Velocity Accuracy (cm/s)	Resolution (ppcm)	Cameras on Target
SSF, T1	mean	6.22	34.93	11.08	147.11	252.44	2.64
	std	14.20	9.63	21.21	8.04	354.72	0.71
	min	0.53	20.91	2.41	56.12	24.06	2
	75%	3.20	42.74	6.48	152.49	247.40	3.00
	max	100.06	58.98	155.81	162.32	2595.25	4
DSF, T1	mean	6.20	30.81	11.23	144.05	640.93	2.68
	std	13.61	7.84	20.61	6.95	360.19	0.63
	min	0.52	20.34	2.84	56.12	26.74	1
	75%	3.50	36.98	6.59	148.74	777.13	3.00
	max	101.00	58.72	152.40	159.04	2932.68	4
SSF, T2	mean	5.52	43.99	11.22	151.41	212.74	2.68
	std	12.71	22.46	22.46	11.30	342.32	0.84
	min	0.47	20.33	2.06	56.12	25.25	2
	75%	3.15	47.70	6.40	155.23	189.48	3.00
	max	100.30	146.31	162.78	188.54	2557.83	5
DSF, T2	mean	5.53	37.92	11.02	148.11	515.59	2.70
	std	12.27	15.86	21.32	9.49	386.28	0.84
	min	0.51	20.02	2.31	56.12	26.99	1
	75%	3.29	42.00	6.44	151.53	690.15	3.20
	max	101.30	107.76	164.31	177.72	2587.40	5
SSF, T3	mean	12.48	55.74	19.56	155.09	219.22	2.48
	std	23.89	54.56	30.89	18.54	302.08	0.92
	min	0.51	20.29	2.36	56.12	48.32	1
	75%	7.72	49.88	15.67	158.08	243.49	3.00
	max	110.71	373.64	142.82	234.54	2451.09	5
DSF, T3	mean	9.11	35.37	14.33	146.87	506.88	2.57
	std	18.20	14.12	25.07	8.97	378.46	0.78
	min	0.61	19.75	2.41	56.12	11.21	1
	75%	4.44	40.39	7.68	150.60	710.26	3.00
	max	108.46	100.77	142.88	176.90	2528.80	5

**Table 6:** DSF performance: $C_{7}^{D}$ or $C_{8}^{D}$ not-working, 1 target following trajectory T2.

Case Study		Position Precision (cm)	Position Accuracy (cm)	Velocity Precision (cm/s)	Velocity Accuracy (cm/s)	Resolution (ppcm)	Cameras on Target
DSF, T2 without $C_{7}^{D}$	mean	5.69	38.94	11.19	148.78	487.20	2.49
	std	12.99	15.53	21.88	9.34	373.27	0.61
	min	0.46	20.02	2.38	56.12	27.74	1
	75%	3.04	42.06	6.31	151.54	663.70	3.00
	max	98.70	107.67	162.16	177.71	2598.42	4
DSF, T2 without $C_{8}^{D}$	mean	5.45	38.23	10.87	148.41	471.25	2.50
	std	12.21	15.75	21.34	9.42	365.58	0.65
	min	0.43	20.03	2.35	56.12	9.55	1
	75%	3.15	42.06	6.45	151.56	614.89	3.00
	max	99.41	107.63	162.61	177.69	2620.06	4

**Table 7:** PTZ parameter selection computational time: 4 targets following trajectory T1.

Case Study	Computational Time (s)	
4 targets	mean	0.229
	std	0.067
	max	0.330

**Table 8:** DSF performance: 1 and 4 targets following trajectory T1.

Case Study		Position Precision (cm)	Position Accuracy (cm)	Velocity Precision (cm/s)	Velocity Accuracy (cm/s)	Resolution (ppcm)	Cameras on Target
DSF, T1	mean	6.20	30.81	11.23	144.05	640.93	2.68
	std	13.61	7.84	20.61	6.95	360.19	0.63
	min	0.52	20.34	2.84	56.12	26.74	1
	75%	3.50	36.98	6.59	148.74	777.13	3.00
	max	101.00	58.72	152.40	159.04	2932.68	4
DSF 4, T1	mean	6.47	33.49	11.50	146.04	448.50	2.54
	std	14.60	8.70	21.67	7.50	364.33	0.67
	min	0.54	20.37	2.38	56.12	16.87	1
	75%	3.35	40.31	6.64	150.42	584.09	3.00
	max	107.39	76.01	156.20	170.34	2718.68	4

**Table 9:** DSF performance: 8 and 12 targets following trajectory T1.

Case Study		Position Precision (cm)	Position Accuracy (cm)	Velocity Precision (cm/s)	Velocity Accuracy (cm/s)	Resolution (ppcm)	Cameras on Target
DSF 8, T1	mean	6.34	33.57	11.31	146.05	358.64	2.65
	std	14.40	9.71	21.40	7.75	374.65	0.64
	min	0.46	20.46	2.37	56.12	20.89	1
	75%	3.32	40.05	6.63	150.68	433.44	3.00
	max	108.73	70.80	154.19	164.52	2740.93	4
DSF 12, T1	mean	6.44	34.61	11.40	146.82	316.11	2.60
	std	14.61	10.02	21.62	8.10	364.13	0.66
	min	0.50	20.53	2.29	56.12	17.72	1
	75%	3.28	41.80	6.62	151.91	380.52	3.00
	max	109.30	78.20	155.42	170.34	2782.13	4

**Table 10:** PTZ parameter selection computational time: 8 targets following trajectory T1.

Case Study	Computational Time (s)	
8 targets	mean	0.472
	std	0.035
	max	0.567

**Table 11:** PTZ parameter selection computational time: 12 targets following trajectory T1.

Case Study	Computational Time (s)	
12 targets	mean	0.780
	std	0.061
	max	0.959

**Table 12:** DSF performance: multiple targets having different velocities.

Case Study		Position Precision (cm)	Position Accuracy (cm)	Velocity Precision (cm/s)	Velocity Accuracy (cm/s)	Resolution (ppcm)	Cameras on Target
DSF 4, 1 m/s	mean	6.47	33.49	11.50	146.04	448.50	2.54
	std	14.60	8.70	21.67	7.50	364.33	0.67
	min	0.54	20.37	2.38	56.12	16.87	1
	75%	3.35	40.31	6.64	150.42	584.09	3.00
	max	107.39	76.01	156.20	170.34	2718.68	4
DSF 4, 2 m/s	mean	22.38	41.31	34.85	201.40	414.55	2.46
	std	39.77	11.41	57.91	12.31	408.15	0.70
	min	0.66	26.39	3.30	73.48	11.82	1
	75%	16.97	47.48	33.84	206.44	554.98	3.00
	max	197.52	105.16	306.46	241.77	3020.25	4
DSF 4, 4 m/s	mean	83.27	54.99	128.47	279.65	330.76	2.39
	std	104.94	21.08	151.46	22.80	383.58	0.76
	min	1.18	35.29	4.70	99.50	18.52	1
	75%	125.24	59.85	193.71	286.78	423.86	3.00
	max	403.85	144.50	603.55	329.85	2492.24	4

**Table 13:** SSF and DSF performances: real-world scenario.

Partition Number		Resolution (ppcm)		Cameras on Target	
		SSF	DSF	SSF	DSF
mean between $P_{2}$ & $P_{3}$	mean	320.37	433.06	2.58	2.50
	std	234.84	246.87	0.37	0.314
	min	64.31	71.25	2	1.77
	max	2147.16	2514.06	3.93	4
$P_{1}$	mean	136.94	348.13	2.72	2.64
	std	244.60	238.72	0.46	0.31
	min	24.22	20.85	2	1.3
	max	269.84	1200.11	4	3
$P_{4}$	mean	74.38	157.40	2.43	2.18
	std	40.78	70.01	0.61	0.37
	min	26.99	36.84	1.5	1.27
	max	269.17	378.41	4	3
$P_{5}$	mean	143.93	335.43	3.35	3.37
	std	82.02	197.68	0.62	0.37
	min	29.80	39.58	2	2
	max	732.08	1290.00	5	4.02

**Table 14:** SSF and DSF performance: most-crowded real-world scenario.

Partition Number		Resolution (ppcm)		Cameras on Target	
		SSF	DSF	SSF	DSF
mean between $P_{2}$ & $P_{3}$	mean	253.45	320.09	2.58	2.52
	std	42.53	46.89	0.14	0.06
	min	179.11	208.7	2.33	2.39
	max	360.30	427.27	2.85	2.64
$P_{1}$	mean	174.11	271.9	2.9	2.7
	std	58.28	51.59	0.15	0.12
	min	101.49	150.9	2.52	2.37
	max	410.27	461.92	3.14	2.97
$P_{4}$	mean	124.13	293.68	2.66	2.63
	std	17.89	94.03	0.17	0.1
	min	99.75	150.9	2.26	2.45
	max	165.76	440.27	2.95	2.85
$P_{5}$	mean	146.31	251.95	2.86	2.69
	std	27.98	49.9	0.16	0.14
	min	101.49	150.9	2.53	2.37
	max	189.61	374.65	3.14	2.97

## Data Availability

Not applicable.
